# Supplementary material for: Exposure assessment of adults living near unconventional oil and natural gas development and reported health symptoms in southwest Pennsylvania, USA
Source: PLoS One. 2020 Aug 18;15(8):e0237325. doi: 10.1371/journal.pone.0237325 (PMC7446921; doi:10.1371/journal.pone.0237325)
Supplement: Attachment — Submitted filename: Review of PONE-D-19-34629.pdf [file pone.0237325.s002.pdf]

Summary of Article
This study uses voluntary health surveys (taken across six years) and data on oil-and-gas (O&G) well locations and annual emissions to identify correlations between reported symptoms and estimates of intensity of exposure to well emissions. Eight counties in southwestern Pennsylvania were the focus. Spatial density of wells within various radii (cumulative well density—CWD) was one measure of exposure intensity, while inverse-distance weighting (IDW) was another measure. Annual well emissions were used in combination with hourly meteorological data in a screening dispersion model to estimate outdoor concentrations at the respondents' houses (CONC), as a third measure of exposure intensity. These estimates of exposure intensity were on an annual basis. Each respondent reported whether they were experiencing any symptoms listed in the survey, and professionals examined the responses to control for symptoms that could be explained by known health issues or other factors. Generalized linear models (GLMs) tested for associations between estimated exposure intensity and total number of symptoms reported by respondents, finding the strongest association with CWD, and a weaker but still statistically significant association with CONC; IDW did not demonstrate a statistically significant relationship to total symptom count. Some observations about effect of sex, smoker status, and drinking-water source were made. Threshold Indicator Taxa Analysis (TITAN) revealed that many individual symptoms were statistically significantly correlated with changes in estimated exposure intensity. While these results do not directly link well activities to reported health complications, they join a larger body of evidence in the literature altogether suggesting that a variety of health issues appear correlated with oil-and-gas activities, at distances beyond the proximity-based setback regulations used in some locations.

Overall Impressions/Summary of Major Comments
This article is well organized and makes a valid contribution to the body of literature on the topic of potential health impacts from oil-and-gas activities.

It is particularly interesting that the correlation with total reported symptoms is stronger with CWD than with IDW, and the authors make a good case for why that is—that being, the authors did not have data on the particulars of each well's activities (beyond location and annual emissions), and assigning higher weights to wells closer to a residence compounds that uncertainty. In my opinion, that points out a notable limitation in the methodology and conclusions of this study, which I believe the authors should be more straightforward in acknowledging. That is, the health concerns reported by residents are correlated only with annual data on well locations and emissions. It is not known if the health issues were transient or longer lasting (which the authors acknowledge), and it is not known exactly what was going on at the well pads within 5 km of their house. We know that wells under development can have highly variable emissions, perhaps by orders of magnitude, and some wells may only be under development for weeks before going into production mode, during which emissions are generally much smaller. The body of literature suggests that higher air concentrations resulting from O&G activities are much more likely to occur during development, and that reports from local residents of health issues and nuisances also tend to peak during development. Therefore, correlating one-time reports of health complaints with annual O&G data is missing an

opportunity to more directly investigate possible connections between health complaints and O&G operations in real time. Can you say if new well development is active and thriving in these counties, mixed with wells in long-term production mode? Are there really no data on which wells were under development vs. in production, with sufficient time resolution to draw closer connections?

I appreciate that the authors used CONC as a third exposure-intensity metric. Though the emissions data are annualized, and the dispersion model is screening-level, this is an important metric because it combines proximity to residence with emission source strength. And if you are asserting that these health-symptom reports may be linked inhaling chemicals emitted from well pads, then emissions and proximity are key to that exposure route. However, you should be clearer in your assumption that the respondents' exposures are entirely at their residence (or at least that's what this intensity metric represents) and that there is full chemical penetration into their home. I also found the "Ambient Air Emissions" methodology section to be rather unclear on a number of fronts, as I discuss in more detail below. This is the section that I advise the most revisions to.

I also appreciate that the authors used several characteristics of respondents as part of the GLM application. However, the discussion of the role of those characteristics in correlating symptom reporting with changes in exposure intensity is non-existent. In my opinion, if you are making observations about statistical differences in males/females, smokers/non-smokers, age, water source, etc., then the discussion on them should be more complete and include speculations about the meaning of those differences. If you are not willing to speculate, then say why.

More specific line-by-line comments are provided below. Some of them are related to my comments above, while others are newly mentioned below (some of them major).

I appreciate you giving me the opportunity to review your work. I hope that my comments are helpful and fair, and I am available to review a revised manuscript should you decide to go that route.

Additional Comments

- "Household" refers to the people in the house. I think in most cases you mean "residence" (i.e., the location of the house).
- In many cases, you use "gas well" as shorthand for "oil and gas well" but it implies by omission that they're not oil wells. Consider just using "well".
- If you estimate concentrations from emissions, then consider if your model names and results discussions should refer to a "concentration model" and "concentration intensity" and "concentration gradient" etc. rather than emissions model, intensity, gradient, etc.
- Not defining duration of symptom (short periods vs long periods of symptom persistence) is a concern in terms of understanding if reported health issues are episodic versus

chronic. Not correlating time of symptom with UOGD activity also weakens assumptions about correlations between well activities and health issues.

- I take some issue with calling the reported symptoms “health effects”, “health impacts”, and similar phrasing. These phrases imply cause (O&G emissions) and effect (itchy eyes, etc.). Perhaps terms like “negative health symptoms” are more appropriate?
- I also think you should be more careful about referring to CDW and IDW as measurements of exposure. They’re metrics of proximity to wells, and that’s it. CONC is closer to an exposure metric, as you attempt to estimate air concentrations of O&G-emitted chemicals, at residences. I think at the least you should acknowledge this, and perhaps then establish that for convenience you will refer to them as metrics of potential exposure intensity (or something like that).

- **Abstract** (minor issues)
    - Lines 26–27: suggest rewording sentence to: “We investigated UOGD density and well emissions and their associations with symptom reporting by residents of southwest Pennsylvania.”
    - Line 28: change “from 2012-2017” to “in 2012–2017” (en dash)
    - Line 31: insert comma after “intensity”
    - Line 34: change “ambient air emissions” to “ambient-air emissions”
    - Line 35: a dispersion model does not quantify emissions
    - Line 41: change “comprised of” to “constituted”
    - Line 42: I think you should change “increased” to “increases”?

- **Introduction** (minor issues)
  This section provides a good though brief review of the literature on potential connections between O&G operations and impacts on human health.
    - Lines 48–49: change “human health risk” to “human-health risk”
    - Line 61: change “number” to “numbers”
    - Lines 67–68: insert comma after the [8] citation, and change “inverse distance weighting” to “IDW”
    - Lines 69–70: I think you should change “well emissions exposure” to “emissions exposure metric”? Also, change “calculate ambient air at the” to “calculate ambient-air concentrations at the”. Change “exposure metric comparison as well, however, their” to “exposure-metric comparison as well, but their”
    - Line 72: I think you should put “[16]” after the Hess citation?
    - Lines 74–75: suggest revising as “...and this analysis—comparing three estimates of exposure, including reported emissions—attempts…”
    - Final sentence starting on Line 78: suggest changing to “The aggregate of methodologies applied here—using statistical modeling to analyze the influence of different exposures on symptom reporting, and applying a technique to identify specific symptoms that might be indicative of exposure—is novel in UOGD research and provides insight into new techniques for studying relationships between health and exposure variables.”

- **Study Sites & Health Outcomes**
  This section provides a good description of the symptom reports and provides confidence that they were screened appropriately.
  - Major Issues
    - Line 90: What does “Appendix A” refer to? The reference doesn’t have an Appendix A, and neither do you? Also, the link provided for reference 18 is broken, I think you’re missing a hyphen between “individual” and “heath”.
  - Minor Issues
    - Line 87: change “Between” to “In”
    - Line 95: I think you should change “Weinberger et al. the” to “Weinberger et al. [19], the”?
    - Line 97: change “oil and gas industry” to “oil-and-gas industry”
    - Line 98: suggest changing “complete the assessment form (n=118). The 118 health assessments” to “acomplete the assessment form (17 excluded). The remaining 118 health assessments”
    - Line 99: change “health care providers” to “health-care providers”, and “occupational health physician” to “occupational-health physician”
    - Line 103: change “one of eight counties” to just “eight counties”
    - Figure 1 caption: remove comma in “Southwestern, PA”; change “Lawrence county” to “Lawrence County”; insert “County” after “Butler”; change the “[20]” citation to “[22]”
    - Figure 1: suggest making county names more readable (move them on top of the well locations?)

- **Cumulative Well Density and Inverse Distance Weighting**
  This section lacks some clarity, as discussed below.
  - Major Issues
    - Line 119: You say that three radii were drawn initially. That implies something changed later…?
    - Line 122: suggest updating this sentence to “Active, unconventional wells for the year of a completed health assessment were plotted within the three radii around the respondent’s home.” Though 5 km was the maximum radius, it’s probably more clear to say within the three radii.
    - Line 123: update this sentence to “A cumulative well density was calculated per respondent for the year of their survey, equal to the total number of wells divided by the radius (in km).” Again, it’s using three radii with 5 km being the max, right?
    - Line 135 about the four residences with wells outside of PA within their 5-km radius (insert hyphen there!): were they also outside of PA for their 1- and 2-km radii? Also, why not throw these respondents out of your

study since you essentially had to remove part of their buffer areas, making their exposure measures underestimates?

- Minor Issues
  - Citation numbering got messed up in a few spots. I think [20] in the first paragraph should be [21], and in the second paragraph [22] should also be [21] while [20] should be [22]. Please check. Also in the second paragraph, I think you should insert “[19]” after the Weinberger citation.
  - Line 120: change “1km” to “1 km” (insert space)
  - Line 126: the “IDW” abbreviation was already established earlier.
  - Line 127: should “qualifying” be “quantifying”? Leading into the next line, change “closer to the respondents’ home” to “closer to a respondent’s home”.
  - Line 128: suggest updating this sentence to “The inverse distance of each well within 1-, 2, and 5-km radii of a residence was calculated, and those values were summed into one IDW score per respondent, per radius, as shown in the following equation:”
  - Lines 132–133: change “respondents’ home, and *n* is the number of wells within the 5 km buffer” to “respondent’s home, and *n* is the number of wells within the radius”

- **Ambient Air Emissions**
  - Major Issues
    - “Ambient Air Concentrations” is a more appropriate section title, as those are what you are deriving in this section.
    - Line 139: should year 2012 been removed from your assessment, given that 25% of the year’s emissions data were unavailable?
    - Line 155: for the Pittsburgh meteorological data, can you speak to their representativeness of conditions across your study area?
    - Line 163: what is the significance of modeled concentrations being less than 10 $\mu g/m^3$, and what was that based on?
    - Lines 171–173: an air concentration is not a “rate of emissions exposure”, it’s a concentration. You can then say (if correct) that you assume that it is the concentration at which residents are exposed (i.e., constant exposure to outdoor concentrations at their residence), and refer to it as an exposure concentration. What is meant by “total, or aggregated, emissions”—the one emissions rate you say earlier that you used in your calculations? Repeating that here makes it sound like there’s something more going on here at the end to determine individual exposure.
    - This section is very difficult to follow. This is due to several factors.
      - There is an inconsistency in terminology that can be easily rectified: you are using emissions of chemicals from well sites (which indeed are ambient air emissions, though “air emissions” is clear enough; and they are reported as rates, not volumes; a

consistent use of emission “rate” rather than “amount” is desired here, too) and meteorological data to estimate concentrations (not “levels” or “emissions concentrations” or “air level value”) of those chemicals in the ambient air at various distances from the well.

- You kindly offer a brief summary of a box-model methodology more fully described in other papers, but the brief summary as it is currently written is inadequate and confusing. Pasquill used five wind-speed categories, along with cloud cover and time of day, to define six stability classes, not 30. I see that your reference [23] (Brown et al., 2019) has a Table 1 that defines 30 stability classes from A1 to D30, but I don’t recall seeing these from Pasquill’s work (certainly correct me if I’m wrong!) and I don’t see how that’s used in concert with Figure 1 of [23] which is the vertical mixing/stability/distance look-up chart showing just the six stability classes. You say you pulled data on wind direction from NOAA, but the box model does not utilize wind direction. Taking a step back from the details, it would be clearer to say (roughly) that the model utilizes atmospheric stability, wind speed, and an assumption about the size of a well-pad facility to estimate the size of a box in which the emissions are well mixed, which in turn is a measure of plume dilution, where the chemical concentration in the box is calculated as emission rate divided by box volume. (Hourly wind speed is used as part of the box volume calculation, right? That’s the “meters of air that pass over a site/minute” stated in [23]?) Then you can march through how you identified each of those parameters (Pasquill stability from hourly data on cloud cover and wind speed from NOAA; an assumed 100-m diameter of well pad; Pasquill assumptions on vertical mixing given stability and horizontal distance; an assumption of constant 300 g/h emissions).
- The relationship between the reference emission rate used in the modeling (300 g/h) and the actual facility emission rates (variable) is not entirely clear. I think you’re telling me that you ran the model to get concentrations per unit emissions at five different distances from a well, based on a high-end metric of hourly concentrations in a year (why 300 g/h and not just 1 g/h?). Then you got a well’s real emissions and multiplied them by the modeled concentration per unit emissions. If that is correct, please consider updating the final paragraph of this section to be more clear about this.
- The use of quadrants around a residence is not clear to me. How does this affect concentration in any way?

○ Minor Issues

        - Line 139: why does it matter that emissions data after December 31, 2017 were unavailable? The health survey data stopped after 2017 anyway.
        - Line 140: insert hyphen: “emissions-inventory data”
        - Line 141: define PM 2.5
        - Line 142: reference [22] should be [21]
        - Lines 144–146: what emission sources weren’t included? You mention this much later, but would be good to mention here, too.
        - Line 154: “that was collected at the Pittsburgh Allegheny County Airport” is more clearly stated as “for the Pittsburgh Allegheny County Airport”
        - Line 156: your study was through 2017, not 2016.
        - Line 162: insert hyphen: “16-km radius”

- **Statistical Analysis**
    - Major Issues
        - Lines 184 and 219: was the threshold of significance 0.5 or 0.05?
        - Final paragraph: what is the purpose of only including symptoms reported 5+ times, and organizing symptoms with frequencies > 10?
    - Minor Issues
        - Line 181: insert comma after “intensity of each exposure”
        - Line 185: insert “for” after “were used”?
        - Line 186: change “a model selection and averaging tool” to “a tool for model selection and averaging”
        - Line 187: change “best fit” to “best-fit”
        - Line 189: change “one hundred” to “100”
        - Line 190: I’m not sure what “to model assessment” means, and why 100 models?
        - Line 194: change “model” to “models”
        - Line 202: insert comma after “emissions”

- **Symptom Reporting Characteristics** (minor issues)
    - Line 227: is the median age better stated as “57 ± 1 standard deviation (SD)”? Note to put a space after ±. If this is the correct expression, then consider also updating lines 230–231 to say “with a mean of 7 ± 7.7 SD”.
    - Lines 229–230: is this sentence saying that some respondents reported 0 symptoms, while one or more respondents reported 36 symptoms, with an average of 7 reported per person? Consider rewording to be more clear.

- **Generalized Linear Models: Symptom Total**
  This section really tees up the rest of the results sections. Therefore, I feel (as laid out below) that it lacks clarity with respect to the statistical presentation, particularly the understandability of Table 2 and what exactly you’re saying about respondent characteristics.
    - Major Issues

- I must admit that Table 2 is difficult to follow. Would a strong statistician understand this? Under a given model, should I be looking to the line restating the model name as the variable, to find statistical significance (e.g., p<0.001 for Cumulative well density-Cumulative Well Density and for Aggregated emissions-Aggregated Emissions, but p=0.316 for IDW Score-IDW Score)? What's the meaning of the "Cumulative Well Density: Age" type rows, and why is there only one of these kinds of rows for the IDW Score model, while three for Aggregated emissions model and five for Cumulative well density model?
- Paragraph starting Line 250: I'm not sure what the second sentence is saying, the part about "occurred between smoker status". It appears to me that there was little-to-no relationship between number of symptoms reported by current/former male smokers and magnitude of emissions, and for males not reporting their smoker status the model actually shows a declining number of reported symptoms and as emissions increased. I think your declarations of these results could use qualifying terms like "generally" or "on average": "Increased symptom reporting also generally occurred…", "Females on average also reported more symptoms than males…", and so on. Change "with" on lien 254 to "between". Final sentence: you probably should say that water source isn't shown in Figure 2, for clarity? However, again going back to my confusion over Table 2, doesn't it show p=0.049 (which is >0.001) for Cumulative Well Density: Water Source, and thus isn't statistically significant?
- Figure 2b's X axis is better labeled as concentration rather than emissions, as covered earlier in my comments.

- Minor Issues
  - First sentence: consider rewording to "Based on the initial GLMs (including demographic variables), models using a 5-km radius for cumulative well density had the lowest AIC value (relative to the 1- and 2-km radii), and 5 km was therefore used as the defining radius for cumulative well density as an exposure variable…"
  - Line 238: change "from 0.05 to 0.73, thus all three" to "from 0.05 to 0.73; thus, all three"
  - Figure 2a's X axis should have a space between 5 and km ("5 km").
  - Line 248: insert "did" before "emissions"
  - Line 250: revise to "In the cumulative-well-density model…"

- **TITAN Analysis**
  This section is generally well written and easy to follow.
  - Major Issues
    - Lines 281–282: range and mean was the same as what? Same on line 295.
    - Figures need y-axis labels

- Minor Issues
    - Lines 264–265: update “along the cumulative well density, IDW, and emissions gradients” to “along gradients of cumulative well density, IDW, and emissions”. Otherwise, proper grammar would suggest that you say “along the cumulative-well-density, IDW, and emissions gradients”, which is fine but some consider to be awkward. Consider this throughout this section (e.g., like 267 “cumulative-well-density gradient” or “gradient of cumulative well density”).
    - Figure 3 discussion: Suggest you introduce the figure on line 266: “Fig 3 shows results for cumulative well density, with the 23 significant symptoms displayed. Itchy or burning eyes…”. Then later on line 270, you can remove the “Of the twenty-three significant symptoms, ” preface and just begin “Roughly 26% of the symptoms were categorized…”.
    - Lines 272–273: What is the meaning of negative associations? Increasing well density leading to decreasing reports of headaches, difficulty speaking, ringing in ears, and rash? That’s odd, isn’t it? It might be worth mentioning your “type-I error” hypothesis here? Earlier on line 266, you when discussing the top three indicator values, you probably should include that they’re positive associations, just to be clear?
    - Lines 272 and 281: should you use “negatively associated” or “inversely associated”?
    - Lines 284–285: maybe you mean “followed by nerves and muscle symptoms and psychological symptoms, which comprised 21% of symptoms each”?
    - Lines 285–286: This sentence might be better as “In addition to headaches, difficulty speaking was also negatively associated with the gradient.”? Similarly, lines 294–295: “In addition to headaches, rash and palpitations were also negatively associated with the gradient.”
    - Lines 298–299: end of sentence might be better stated as “with psychological symptoms and nerve and muscle symptoms each at 20%.”?

- **Discussion** (minor issues)
  Overall this is a great section that does a good job putting the study results into context.
    - Lines 309, 314, 318, and 323: “human-health standpoint”, “human-health symptoms”, “human-health impacts”, “human-health metrics” (insert hyphen)
    - Line 311: I’m not 100% certain but I think “further” should be “farther”?
    - Line 314: change “non-UOGD” to “not UOGD”
    - Line 320: remove “that”
    - Line 322: change “does” to “do”
    - Line 325: “5-km” (insert hyphen)
    - Line 334: change “was” to “were”
    - Lines 335–336: “exposure-magnitude impacts (insert hyphen)

- Line 346: I think the “which may lead to underreporting of impacts to health across the literature” should be bounded by commas
- Lines 351–352: consider changing “in each model” to “in each of our models” for clarity
- Lines 365–366: consider “related to the gradients of cumulative well density, IDW, and emissions, respectively”, rather than “related to the cumulative well density, IDW, and emissions gradient respectively”. Similarly later on line 370: “associated with gradients of cumulative well density, IDW, and emissions, respectively, which contributes”
- Last sentence: why would these aspects lead to underestimates of emissions?

- **Limitations & Recommendations** (minor issues)
  Like the Discussion section, this is well written and generally has the appropriate content.
  - Lines 375–376: “health-reporting data” (insert hyphen)
  - Line 378: should the Tustin and Rabinowitz citations have numbered indicators (and Elliot on line 379, Rabinowitz on line 385)?
  - Lines 391–392: consider revising sentence to “In future studies, other health indicators or metrics such as blood pressure, heart rate, and the number of days a symptom persisted could provide a more in-depth analysis and help define the severity of the symptoms experienced.”
  - Line 400: change to “The air-and-exposure screening model” (insert hyphens)

- **Conclusion** (minor issues)
  - Line 408: remove extra space before “an IDW metric”
  - Line 410: “detrimental health” is awkward, do you mean health complications?
  - Line 414: insert “the” before “relatively young age”
  - Line 416: change “but do raise” to “but they do raise”
  - Line 418: change “oil and gas industry” to “oil-and-gas industry”
  - Lines 418–419: suggest rewording to “Our study suggests that the narrow consideration of exposure risk used in some locations—based only on proximity to an individual well—may warrant revision to assess the contribution of the growing density of wells in and around communities at 5-km scales.”
