# Supplementary material for: Exposure assessment of adults living near unconventional oil and natural gas development and reported health symptoms in southwest Pennsylvania, USA
Source: PLoS One. 2020 Aug 18;15(8):e0237325. doi: 10.1371/journal.pone.0237325 (PMC7446921; doi:10.1371/journal.pone.0237325)
Supplement: Attachment — Submitted filename: PONE-D-19-34629R1 - Reviewer 3 Comments.pdf [file pone.0237325.s004.pdf]

Reviewer #3 review of the first revision of **Exposure assessment of adults living near unconventional oil and natural gas development and reported health symptoms in southwest Pennsylvania, USA**, Blinn et al. (PONE-D-19-34629R1)

Thank you for the opportunity to review your revised manuscript. Your revisions have satisfactorily addressed most of my concerns from my review of your initial submission. As with my first review, I currently have numerous minor concerns related to proofreading. Aside from those, I have a small number of less minor concerns related to your methodology, and one related to your conclusions, that should be addressed within the paper before I can recommend publication.

All comments below are minor, except where you see "MAJOR COMMENT".

I will be happy to review an additional revision.

Abstract

- Line 39 - remove "of" before "50%"
- Line 40 - change "grouping" to "groupings"

Introduction

- Line 51 - remove hyphen in "human-health"
- Lines 62-63 - think you mean "Which exposure measure(s) is the best predictor…" or "Which exposure measures are the best predictors…"
- Line 76 - did you mean "different" instead of "difference"?
- **MAJOR COMMENT**: Lines 98-99 - it's not clear why your analysis was restricted to the eight counties

Materials and Methods

Exposure Measures

Cumulative Well Density and Inverse Distance Weighting

- Line 116 - change "were plotted" to "was plotted"
- Line 119 - remove "was completed"

Annual Emissions Concentration

- Line 136 - change "rate" to "rates"
- Line 143 - you mean "concentrations" not "emissions"
- Lines 146, 147, 151 - "down-wind" "downwind" - choose one
- **MAJOR COMMENT**: Line 156 - I still don't understand what part wind direction played in your modeling.
- **MAJOR COMMENT**: Line 159 - I still think you don't adequately address in the paper the representativeness of the data at the Pittsburgh airport to the region at large. I don't think it's the only hourly station in the eight-county area that reports the variables you use. Why did you use this one station?
- Line 162 - change "was" to "were"
- Line 163 - remove "yearly" as it implies annual-average weather, when in fact you used hourly weather data

- Line 172 - change “respondents’” to “respondent’s” and “was” to “were”
- **MAJOR COMMENT**: Paragraph beginning Line 173 - Your methodology treats the trajectory of each well’s plume equally when summing the quadrant concentrations together. In fact, a wind rose analysis would tell you how often winds blew from each quadrant toward each residence, allowing you to weight the concentrations from some quadrants more than others. You should either address this limitation or correct for it.
- Line 173 - suggest changing “surround” to “are ubiquitous around”
- Line 176 - I think you mean “in which quadrant the well was located relative to the residence”?
- Line 182 - change “of their survey and was used” to “of their survey, which was used”

Statistical Analysis

- Line 186 - should “compared” be changed to “made”?
- Line 201 - should “to generation” be “for determining”?
- Line 213 - “a species” is singular but “taxa” is plural - change “taxa” to “taxon”?
- Line 214 - change “are” to “is”
- Line 223 - change “uses” to “used”
- Line 232 - change “z score” to “z-score”

Results

Generalized Linear Models: Symptom Total

- Line 248 - change “measures” to “measure”
- I don’t think Figure 2 is cited in the text?

Discussion

- Lines 306, 314 - remove hyphen in “human-health”
- **MAJOR COMMENT**: Line 311 - you only evaluated wells within 5 km of a residence, so how did you detect effects beyond that?
- Line 333 - remove “primarily”, all respondents were concerned about UOGD exposure
- Line 342 - end of line, change “was” to “were”
- Line 343 - remove hyphen in “exposure-magnitude”

Limitations & Recommendations

- Line 395 - I believe development and production are the ONLY UOGD stages, and I believe the literature supports that emissions from development tend to be higher than those from production
- **MAJOR COMMENT**: Line 401 - your model actually does account for day-to-day weather patterns, as it utilizes hourly weather data. Do you mean that your model does not correct for differences in weather observations at residences relative to the weather station site?

Conclusion

- Line 406 - I think you mean “emissions concentrations”. Air dispersion modeling does not quantify emissions, it uses them to quantify concentrations. Also, your study is not the first to use dispersion modeling for UOGD purposes.
- Line 411 - remove hyphen in “human-health”
- Lines 417-418 - I think you want to remove “in and around communities at”
- Line 419 - remove hyphen in “residence-level”

- Line 420 - remove hyphen in “human-health”

S2 Appendix

- Line 577 - change “CDW” to “CWD”
