# Supplementary material for: Exposure assessment of adults living near unconventional oil and natural gas development and reported health symptoms in southwest Pennsylvania, USA
Source: PLoS One. 2020 Aug 18;15(8):e0237325. doi: 10.1371/journal.pone.0237325 (PMC7446921; doi:10.1371/journal.pone.0237325)
Supplement: Attachment — Submitted filename: Response Letter.docx [file pone.0237325.s003.docx]

Board of editors	6 March 2020

*PLOS ONE*

Dear colleagues,

Thank you for your feedback concerning our recently submitted manuscript, “*Exposure Assessment of Adults Living Near Unconventional Oil and Natural Gas Development and Reported Health Symptoms in Southwest Pennsylvania, USA”* for potential publication in *PLOS ONE.* Below you will find a response table with each reviewer’s comments and how we addressed the comments in the revised manuscript. Page numbers are provided for your convenience. We wanted to highlight other changes in the manuscript that are not clearly addressed in the table. First, we noticed in our calculations that the exposure measure inverse distance weighting (IDW) was incorrectly calculated compared to what we stated in the methods. Our methods stated computing a 1/distance (km) whereas our calculations calculated a 1/distance^2^ (km^2^). We corrected the formula to match the former, a similar metric to what was applied in Stacey et al. [3]. This correction and the simplification of our statistical modeling resulted in IDW becoming a statistically significant predictor of symptom total. The results and discussion were updated accordingly. We also wanted to highlight a modification in author affiliation. As shown in the revised manuscript, the primary and only affiliation for Lydia Greiner should be the Southwest Pennsylvania Environmental Health Project. Lastly, two pieces of supplemental materials have been added to this submission to be included as an appendix for this manuscript.

Health data cannot be shared publicly because some of the data we collect is in rural areas with sparse population. In areas of sparse population, it may be possible to identify participants using data such as GIS coding. Data are available from the Environmental Health Project Institutional Data Access / Ethics Committee (contact via EHP Sara Rankin 724.260.5504) for researchers who meet the criteria for access to confidential data.

Thank you for your again for your consideration and thoughtful responses to our work.

Sincerely,

Hannah Blinn

Southwest PA Environmental Health Project

hnblinn@gmail.com;

hblinn@environmentalhealthproject.org

(724) 841-1239

Ryan Utz

rutz@chatham.edu

David Brown

dbrown@environmentalhealthproject.org

Lydia Greiner

lgreiner@environmentalhealthproject.org

| **Comments** | **Response and Action Taken to Comments** | **Line numbers in revised manuscript where action was taken** |
| --- | --- | --- |
| ***Reviewer 1*** | | |
| **Overall Comments** | | |
| This is a very interesting and well written paper using a technique that is somewhat new for addressing the subject. given that much has been written on the topic, I would suggest stressing your use of statistical modeling to try to better understand the relationships. While you use a convenience sample, the focus should be on testing the model. You acknowledge the limitations of your study, which is refreshing and helpful to the reader. | This has been addressed. | 57-78 |
| ***Reviewer 2*** | | |
| **Overall Comments** | | |
| This manuscript looks at the relationship between three UOGD exposure metrics and symptoms among a population of 135 individuals in SW Pennsylvania who approached the Environmental Health Project given their concerns about UOGD. The study adds new techniques, including TITAN borrowed from ecology. They also estimate exposure to UOGD emissions at participant residence, which has been done rarely in the UOGD literature. Poisson models are used to assess associations between the exposure metrics and symptom counts. Models are inappropriately adjusted given the very small sample size and therefore extrapolate beyond the support of the data. Further, the study far oversteps its results in the discussion section | The model selection tool used for the glm analysis adjusts for the small sample size using a corrected Akaike information criterion (AIC) value, also known as an AICc value. We decided to simplify the glm models for this analysis and remove all interaction terms to only look at main effect variables. Doing so, we can discuss how each metric was related to symptom total after accounting for the other covariates. We have modified the discussion to highlight the issue of small sample size and how that may have influenced our results. | 196-206  327-3329 |
| **Major Concerns** | | |
| 1. The Hess et al. 2019 study had major flaws and I urge you not to cite it. If you want to continue citing it, please describe many of its limitations as discussed by Buonocore et al in their letter to the editor published this month. Koehler et al. also discuss exposure metrics in ES&T in 2018. | This has been addressed and the study removed. | NA |
| 2. The number of excluded surveys is very high (~48%). It would be helpful to break this down by reason for excluding the survey, e.g., did most excluded surveys result because the participant did not fully fill it out or because they lived out of state? If most surveys excluded because of incompletion, could you consider doing a sensitivity analysis with this excluded group? | Only 23% of survey participants were excluded from the starting sample of 135, as our n for analysis was 104 adults. More detail on exclusion numbers was included. | 91-101 |
| 3. I am confused about the first metric. “A cumulative well density was calculated per respondent for the year their survey was completed by taking the total number of wells divided by 5 km.” Wouldn’t you either count the total number of wells within 5km and use that as the exposure or divide the total number by pi*5km*5km (true density of wells). I can’t think of a time you would divide by 5km. | This has been addressed and updated in the calculations and text. | 118-120 |
| 4. Please explain why only a single station was used to derive wind speed and direction. This seems to be a major limitation of the model, given that the study area is over 100km wide. Wind could be dramatically different across this area. If you have reason to believe otherwise, please include. Also, why were emissions of 4 pollutants summed when they can have quite different health effects? | This has been addressed. We did not have day to day weather data for each residence in our study. Using annually weather data from a local weather station at the Allegheny County Airport was deemed appropriate by the research team since annual averages are more generalizable across airports in the region, compared to day to day values that would certainly fluctuate. We have included this into the limitations section.  You are correct, each pollutant can have different health effects. For this exploratory study, we chose to combine them. Future work will examine individual pollutants. | 159-162 |
| 5. What is a model selection and averaging tool? Which demographic variables were considered? In addition, you have a relatively small sample size (N = 135) and it appears you run double stratified models (sex and smoking). This means some models have <33 observations. Given a rule of thumb to have 15 observations for each independent variable, you could really only include the exposure and a single confounding variable in these models. Otherwise, you are extrapolating far beyond your data. Therefore, I am not confident in the current modeling strategy. Further, did symptoms really follow a Poisson distribution or was 0-inflation required? | The model selection tool uses AICc, a corrected AIC value for small sample sizes. The tool is used to run all the potential statistical models and determine the best fitting models per the AIC values. This does not mean random subsets of data were generated. We simplified the models by removing the interaction terms as possibilities in the final model. 0-inflation was not required for our data as only 15% of the sample reported no symptoms. | 196-209 |
| 6. I commend the authors for trying something new but the TITAN analysis is fairly confusing. Consider adding more information to the appendix how why this strategy was selected and how it was implemented, perhaps with a toy example | We have added supplemental documentation by the way of sample code and a real-world example of TITAN being used in ecology. | 223-225  S2 Appendix & submitted R code |
| 7. Lines 327-330: this interpretation of the findings is extremely strong. You have a highly selected sample (people that were worried about UOGD) and a tiny sample size (<200 people). Claiming you have identified which metric to use is over-interpreting your results. Same issue lines 357-360. Tustin et al. had a much larger sample of people who did not enter the study based on UOGD concerns. Therefore, you are asking/answering vastly different questions. | This has been addressed. We softened the language and discussed results relative to the characteristics of our sample being small and made up of concerned individuals. We clarified that Tustin et al. did indeed have a larger sample size and therefore isn’t directly comparable. | 324-338  363-365 |
| **Minor Concerns** | | |
| Line 69: should this read ambient air pollution? | This has been addressed. Throughout the text we have standardized this and similar phrases to “annual emissions concentration” or AEC | NA |
| Figure 1 add year of active wells to the figure caption | This has been addressed. | 104 |
| Please upload higher res figures, these are very difficult to read | Figures were submitted separately to the Journal. When they were included in the PDF submission, the quality was reduced. The Journal will have the highest resolution photos we can provide. | NA |
| Figure 3: why are bars different widths? If this indicates something, please make clearer what | This has been addressed. Figure 3 caption explains that the width of the bar is related to the frequency of that symptom being recorded. See the legend on the top right of the image. | 278 |
| Lines 134-136, you say you don’t have data on wells outside PA but then contradict yourself by saying four residences have wells outside PA. If you know about these wells, why not include? | Fractracker.org provides a mapping tool of wells outside of PA but does not give Latitude/Longitude of these wells or provide a downloadable dataset for us to map on our own. We could type in respondent’s addresses and see that there may be wells within their buffer but could not apply this data to what we did in ArcGIS.  We only used PA gas wells so removed reference to wells outside of the state since we are not able to quantify their emissions, and yes, this would underestimate their exposure. Furthermore, only 7 out of 104 subjects we assessed were near enough to state borders to result in a reduced areal extent, and these possessed sample areas >50% within PA. | 130-131 |
| Line 163: and to be consistent with your other exposure metrics using 5km? | This has been addressed. | 117 |
| “An alpha level of <= 0.5 was used as a threshold for significance in both tests” do you mean 0.05? | This has been addressed. | 193 |
| First line of results: it wasn’t just a convenience sample but a sample of people reporting issues with UOGD, right? | This has been addressed and more language about the sample characteristics has been added. | 237 |
| What does median age of +/-1 mean? | This has been addressed. | 238 |
| Smoking status was never mentioned in methods, please add. | This has been addressed. | 199 |
| Results: what was total number of symptoms queried? | 779 | NA |
| Table 1: how many people included in this table? | This has been addressed. | 243 |
| Line 238: which correlation coefficient used? | Pearson – this has been addressed in the text. | 246 |
| How was age modeled? Continuous? What other demographics were considered? | Continuous – this has been addressed in the text. | 249-250 |
| Lines 316-317 see Koehler et al. 2018 re: compressors | This has been addressed. | 318 |
| ***Reviewer 3*** | | |
| **Overall Comments** | | |
| It is particularly interesting that the correlation with total reported symptoms is stronger with CWD than with IDW, and the authors make a good case for why that is—that being, the authors did not have data on the particulars of each well’s activities (beyond location and annual emissions), and assigning higher weights to wells closer to a residence compounds that uncertainty. In my opinion, that points out a notable limitation in the methodology and conclusions of this study, which I believe the authors should be more straightforward in acknowledging. That is, the health concerns reported by residents are correlated only with annual data on well locations and emissions. It is not known if the health issues were transient or longer lasting (which the authors acknowledge), and it is not known exactly what was going on at the well pads within 5 km of their house. We know that wells under development can have highly variable emissions, perhaps by orders of magnitude, and some wells may only be under development for weeks before going into production mode, during which emissions are generally much smaller. The body of literature suggests that higher air concentrations resulting  from O&G activities are much more likely to occur during development, and that reports from local residents of health issues and nuisances also tend to peak during development. Therefore, correlating one-time reports of health complaints with annual O&G data is missing an opportunity to more directly investigate possible connections between health complaints and  O&G operations in real time. Can you say if new well development is active and thriving in these counties, mixed with wells in long-term production mode? Are there really no data on which wells were under development vs. in production, with sufficient time resolution to draw closer connections? | We have added language in several sections to be clearer about these specific limitations. We acknowledge the annual emissions concentration does not account for day to day exposure at the household, given the different stages of well development have different lengths and emissions amounts. We added additional citations to support this claim. Any inclusion of well pad activity or development would have been an estimate on our part, as we do not have access to the start and end date of the various activities that occur on the well. Our dataset only including the spud date for the well. We do know that in these counties, wells are continuously being drilled and same are being re-fracked. That is not tracked on a public database for us to use. | 60-61  159-162  180-182  333-343  379-387  391-397 |
| And if you are asserting that these health-symptom reports may be linked inhaling chemicals emitted from well pads, then emissions and proximity are key to that exposure route. However, you should be clearer in your assumption that the respondents’ exposures are entirely at their residence (or at least that’s what this intensity metric represents) and that there is full chemical penetration into their home. I also found the “Ambient Air Emissions” methodology section to be rather unclear on a number of fronts, as I discuss in more detail below. This is the section that I advise the most revisions to. | We added additional language to add clarity to this assumption and to be clear the exposure metric is calculated at the residence. | 180-182  333-338  416-419 |
| I also appreciate that the authors used several characteristics of respondents as part of the GLM application. However, the discussion of the role of those characteristics in correlating symptom reporting with changes in exposure intensity is non-existent. In my opinion, if you are making observations about statistical differences in males/females, smokers/non-smokers, age,  water source, etc., then the discussion on them should be more complete and include  speculations about the meaning of those differences. If you are not willing to speculate, then say why. | The adjustments we made to simplifying the glm models are meant to also address this point and we added language to the discussion about how our models do account for the covariates like sex, age, and gender. Since we are controlling for the covariates, our discussion focuses on the relationship between exposure metrics and symptom total only (see revised Table 2). | Starting at 245 under “Generalized Linear Models: Symptom Total”.  327-329 |
| **Major Concerns** | | |
| **Study Sites & Health Outcomes** |  |  |
| Line 90: What does “Appendix A” refer to? The reference doesn’t have an Appendix A, and neither do you? Also, the link provided for reference 18 is broken, I think you’re missing a hyphen between “individual” and “heath”. | An appendix has been added and supplemental materials provided separately. | S1 Appendix |
| **Cumulative Well Density and Inverse Distance Weighting** |  |  |
| Line 119: You say that three radii were drawn initially. That implies something changed later…? | This has been addressed. For this study, we only chose to look at wells within a 5-km buffer. The 1-km and 2-km buffers were initially included in the text to explain why we ended up choosing 5 km from a statistical approach (comparison of AIC value between buffer distances and CMD), but we have elected to instead site Elliot et al. (2019) as they also looked at a 5-km buffer range. We removed reference to the smaller buffers as we did not run glm analyses on these distances. | 118-120 |
| Line 122: suggest updating this sentence to “Active, unconventional wells for the year of a completed health assessment were plotted within the three radii around the respondent’s home.” Though 5 km was the maximum radius, it’s probably more clear to say within the three radii. | This has been addressed. | 118-120 |
| Line 123: update this sentence to “A cumulative well density was calculated per respondent for the year of their survey, equal to the total  number of wells divided by the radius (in km).” Again, it’s using three radii with 5 km being the max, right? | This has been addressed. | 118-120 |
| Line 135 about the four residences with wells outside of PA within their 5-km radius (insert hyphen there!): were they also outside of PA for their 1- and 2-km radii? Also, why not throw these respondents out of your study since you essentially had to remove part of their buffer areas, making their exposure measures underestimates? | We only used PA gas wells so removed reference to wells outside of the state since we are not able to quantify their emissions, and yes, this would underestimate their exposure. We added information to describe the percent of these participant’s 5-km buffer that was in West Virginia. | 130-131 |
| **Ambient Air Emissions** |  |  |
| “Ambient Air Concentrations” is a more appropriate section title, as those are what you are deriving in this section. | This has been addressed. Annual Emissions Concentration was the phrase chosen to standardize how we referred to this measure. Thank you for your input on this. | 133 |
| Line 139: should year 2012 been removed from your assessment, given that 25% of the year’s emissions data were unavailable? | It is our understanding that all 2012 data was reported to the PA DEP. Given the industry uses algorithms to estimate their yearly emissions, we question whether they would adjust the formula to exclude months from their calculations. If confusion is coming from Brown et al.’s statement “Since March 31, 2012, owners and operators of natural gas production and processing operations have been required to report air emissions to the PA DEP…” we want to point out that this statement does not reflect a belief that only data after March 2012 was included.  Brown DR, Greiner LH, Weinberger BI, Walleigh L, Glaser D. Assessing exposure to unconventional natural gas development: using an air pollution dispersal screening model to predict new-onset respiratory symptoms. Journal of Environmental Science and Health, Part A. 2019 Dec 6;54(14):1357-63. | NA |
| Line 155: for the Pittsburgh meteorological data, can you speak to their representativeness of conditions across your study area? | This has been addressed. We did not have day to day weather data for each residence in our study. Using annually weather data from a local weather station at the Allegheny County Airport was deemed appropriate by the research team since annual averages are more generalizable across airports in the region, compared to day to day values that would certainly fluctuate. We have included this into the limitations section. | 159-162  392-393 |
| Line 163: what is the significance of modeled concentrations being less than 10 μg/m 3 , and what was that based on? | This has been removed. The rational that we were keeping a buffer of 5-km across our metrics serves as a clearer justification. | NA |
| Lines 171–173: an air concentration is not a “rate of emissions exposure”, it’s a concentration. You can then say (if correct) that you assume that it is the concentration at which residents are exposed (i.e., constant exposure to outdoor concentrations at their residence), and refer to it as an exposure concentration. What is meant by “total, or aggregated, emissions”—the one emissions rate you say earlier that you used in your calculations? Repeating that here makes it sound like there’s something more going on here at the end to determine individual exposure. | This has been addressed. | See section “Annual Emissions Concentration” starting on line 133 |
| There is an inconsistency in terminology that can be easily rectified: you are using emissions of chemicals from well sites (which indeed are ambient air emissions, though “air emissions” is  clear enough; and they are reported as rates, not volumes; a consistent use of emission “rate” rather than “amount” is desired here, too) and meteorological data to estimate concentrations (not “levels” or “emissions concentrations” or “air level value”) of those chemicals in the ambient air at various distances from the well. | This has been addressed. Emissions concentration is the phrase we selected to replace the varying ways we previously were describing it. | See section “Annual Emissions Concentration” starting on line 133 |
| You kindly offer a brief summary of a box-model methodology more fully described in other papers, but the brief summary as it is currently written is inadequate and confusing. Pasquill used five wind-speed categories, along with cloud cover and time of day, to define six stability classes, not 30. I see that your reference [23] (Brown et al., 2019) has a Table 1 that defines 30 stability classes from A1 to D30, but I don’t recall seeing these from Pasquill’s work (certainly correct me if I’m wrong!) and I don’t see how that’s used in concert with Figure 1 of [23] which is the vertical mixing/stability/distance look-up chart showing just the six stability classes. You say you pulled data on wind direction from NOAA, but the box model does not utilize wind direction. Taking a step back from the details, it would be clearer to say (roughly) that the model utilizes atmospheric stability, wind speed, and an  assumption about the size of a well-pad facility to estimate the size of a box in which the emissions are well mixed, which in turn is a measure of plume dilution, where the chemical concentration in the box is calculated as emission rate divided by box volume. (Hourly wind speed is used as part of the box volume calculation, right? That’s the “meters of air that pass over a site/minute” stated in [23]?) Then you can march through how you identified each of those parameters (Pasquill stability from hourly data on cloud cover and wind speed from NOAA; an assumed 100-m diameter of well pad; Pasquill assumptions on vertical mixing given stability  and horizontal distance; an assumption of constant 300 g/h emissions). | This has been addressed. We believe the confusion was coming from the use of stability classes and stability conditions, where they are properly called stability classes and meteorological conditions, based on solar radiation, that define correlated stability classes. In Brown et al. (2019), five wind speeds were used alongside 6 meteorological conditions to create the 30 correlated stability classes that we applied to hourly weather data from the Allegheny County Airport (their Table 1). Pasquill’s original correlated chart shows 5 meteorological conditions, but Brown et al. 2019 added a fourth daytime insolation category.  Brown DR, Greiner LH, Weinberger BI, Walleigh L, Glaser D. Assessing exposure to unconventional natural gas development: using an air pollution dispersal screening model to predict new-onset respiratory symptoms. Journal of Environmental Science and Health, Part A. 2019 Dec 6;54(14):1357-63. | 144-162 |
| The relationship between the reference emission rate used in the modeling (300 g/h) and the actual facility emission rates (variable)  is not entirely clear. I think you’re telling me that you ran the model to get concentrations per unit emissions at five different distances  from a well, based on a high-end metric of hourly concentrations in a year (why 300 g/h and not just 1 g/h?). Then you got a well’s real emissions and multiplied them by the modeled concentration per unit emissions. If that is correct, please consider updating the  final paragraph of this section to be more clear about this. | This has been addressed. You are correct, the 300 g/h is arbitrary as any hypothetical emissions number, above zero, would give the concentration value at each distance. The model was run to determine the concentration at each distance from the well. When we determined the distance between the well and a home, we took that concentration value, multiplied it by the emissions rate from the DEP dataset, and then divided by 300 to get what is estimated to be the emissions concentration at that residence µg/m^3^. | 163-172 |
| The use of quadrants around a residence is not clear to me. How does this affect concentration in any way? | This has been addressed. We factor in what directional quadrant the well is from the home and the appropriate 90^th^ percentile concentration value for that quadrant and the distance from the home is chosen and used to calculate the estimate emissions from a given well. Then we add the concentrations calculated from the North, South, East, and West into one AEC value. The quadrants are a step in the calculation. | 173-182 |
| **Statistical Analysis** |  |  |
| Lines 184 and 219: was the threshold of significance 0.5 or 0.05? | This has been addressed. | 193 |
| Final paragraph: what is the purpose of only including symptoms reported 5+ times, and organizing symptoms with frequencies > 10? | We used these exclusion parameters to remove symptoms that are too infrequent to do statistical analysis with. The numbers are arbitrary and simply provide a way for us cut out infrequencies. | 228-230 |
| **Generalized Linear Models: Symptom Total** |  |  |
| I must admit that Table 2 is difficult to follow. Would a strong statistician understand this? Under a given model, should I be looking to the line restating the model name as the variable, to find statistical significance (e.g., p<0.001 for Cumulative well density-Cumulative Well Density and for Aggregated emissions-Aggregated Emissions, but p=0.316 for IDW Score-IDW Score)? What’s the meaning of the “Cumulative Well Density: Age” type rows, and why is there only one of these kinds of rows for the IDW Score model, while three for Aggregated emissions model and five for Cumulative well density model? | The removal of the interaction terms (Cumulative Well Density: Age) were removed to simplify these models. Table 2 has been updated to reflect these changes. Removing the interaction terms and adjusting the IDW metric as explained in the cover letter let to the IDW metric to be a significant predictor in the study and this has been updated throughout the text.  In the table, the name of the model is given in italics. That is identifying with exposure model was being testing against symptom total (while adjusting for our demographic variables). When you read the table, you will see Cumulative Well Density statistically significantly predicted Symptom Total while adjusting for covariates sex, age, and gender. The model was significant at a p<0.001. | Citation – 230  Table 2 – 256 |
| Paragraph starting Line 250: I’m not sure what the second sentence is saying, the part about “occurred between smoker status”. It appears to  me that there was little-to-no relationship between number of symptoms reported by current/former male smokers and magnitude of emissions, and for males not reporting their smoker status the model actually shows  a declining number of reported symptoms and as emissions increased. I think your declarations of these results could use qualifying terms like  “generally” or “on average”: “Increased symptom reporting also generally occurred…”, “Females on average also reported more symptoms than males…”, and so on. Change “with” on lien 254 to “between”. Final sentence: you probably should say that water source isn’t shown in Figure 2, for clarity? However, again going back to my confusion over Table 2, doesn’t it show p=0.049 (which is >0.001) for Cumulative Well Density: Water Source, and thus isn’t statistically significant? | This has been addressed by simplifying the models to only show main effects. See Table 2. | NA – text was omitted |
| Figure 2b’s X axis is better labeled as concentration rather than emissions, as covered earlier in my comments. | This has been addressed. | NA |
| **TITAN Analysis** |  |  |
| Lines 281–282: range and mean was the same as what? Same on line 295. | This has been addressed and the confusing text removed to simplify the presentation of the results. | NA |
| Figures need y-axis labels | This has been addressed. | NA |
| **Minor Concerns** | | |
| “Household” refers to the people in the house. I think in most cases you mean “residence” (i.e., the location of the house). | This has been addressed. | NA – updated throughout text |
| In many cases, you use “gas well” as shorthand for “oil and gas well” but it implies by omission that they’re not oil wells. Consider just using “well”. | This has been addressed. | NA – updated throughout text |
| If you estimate concentrations from emissions, then consider if your model names and  results discussions should refer to a “concentration model” and “concentration intensity” and “concentration gradient” etc. rather than emissions model, intensity, gradient, etc. | This has been addressed. | NA – updated throughout text |
| Not defining duration of symptom (short periods vs long periods of symptom persistence) is a concern in terms of understanding if reported health issues are episodic versus chronic. Not correlating time of symptom with UOGD activity also weakens assumptions about correlations between well activities and health issues. | This has been addressed. | 380-382 |
| I take some issue with calling the reported symptoms “health effects”, “health impacts”,  and similar phrasing. These phrases imply cause (O&G emissions) and effect (itchy eyes, etc.). Perhaps terms like “negative health symptoms” are more appropriate? | This has been addressed. Negative health symptoms used instead. | NA – updated throughout text |
| I also think you should be more careful about referring to CDW and IDW as measurements of exposure. They’re metrics of proximity to wells, and that’s it. CONC is closer to an exposure metric, as you attempt to estimate air concentrations of O&G-emitted chemicals, at residences. I think at the least you should acknowledge this, and perhaps then establish that for convenience you will refer to them as metrics of potential exposure intensity (or something like that). | This has been addressed and we clarify that CDW and IDW are proximity in the introduction but will be referred to as exposure metrics. In the discussion we re-discuss this distinction. | 60-61  333-336 |
| **Abstract** |  |  |
| Lines 26–27: suggest rewording sentence to: “We investigated UOGD density and well emissions and their associations with symptom reporting by residents of southwest Pennsylvania.” | This has been addressed. | 24-25 |
| Line 28: change “from 2012-2017” to “in 2012–2017” (en dash) | This has been addressed. | 26 |
| Line 31: insert comma after “intensity” | This has been addressed. | NA – text modified or removed |
| Line 34: change “ambient air emissions” to “ambient-air emissions” | This has been addressed. | NA – text modified or removed |
| Line 35: a dispersion model does not quantify emissions | This has been addressed. | 34-35 |
| Line 41: change “comprised of” to “constituted” | This has been addressed. | 39 |
| Line 42: I think you should change “increased” to “increases”? | This has been addressed. | 41 |
| **Introduction** |  |  |
| Lines 48–49: change “human health risk” to “human-health risk” | This has been addressed. | 46 |
| Line 61: change “number” to “numbers” | This has been addressed. | 59 |
| Lines 67–68: insert comma after the [8] citation, and change “inverse distance weighting” to “IDW” | This has been addressed. | 67 |
| Lines 69–70: I think you should change “well emissions exposure” to “emissions exposure metric”? Also, change “calculate ambient air at the” to “calculate ambient-air concentrations at the”. Change “exposure metric comparison as well, however, their” to “exposure-metric comparison as well, but their” | This has been addressed. | 68-69 |
| Line 72: I think you should put “[16]” after the Hess citation? | This has been addressed and the study was removed. | NA – text modified or removed |
| Lines 74–75: suggest revising as “...and this analysis—comparing three estimates of exposure, including reported emissions—attempts…” | This has been addressed. | 71-72 |
| Final sentence starting on Line 78: suggest changing to “The aggregate of methodologies applied here—using statistical modeling to analyze the influence of different exposures on symptom reporting, and applying a technique to identify specific symptoms that might be indicative of exposure—is novel in UOGD research and provides insight into new techniques for studying relationships between health and exposure variables.” | This has been addressed. | 75-78 |
| **Study Sites & Health Outcomes** |  |  |
| Line 87: change “Between” to “In” | This has been addressed. | 83 |
| Line 95: I think you should change “Weinberger et al. the” to “Weinberger et al. [19], the”? | This has been addressed. | 87,91 |
| Line 97: change “oil and gas industry” to “oil-and-gas industry” | This has been addressed. | 96 |
| Line 98: suggest changing “complete the assessment form (n=118). The 118 health assessments” to “acomplete the assessment form (17 excluded). The remaining 118 health assessments” | This has been addressed. | 97-101 |
| Line 99: change “health care providers” to “health-care providers”, and “occupational health physician” to “occupational-health physician” | This has been addressed. | 92 |
| Line 103: change “one of eight counties” to just “eight counties” | This has been addressed. | 100 |
| Figure 1 caption: remove comma in “Southwestern, PA”; change “Lawrence county” to “Lawrence County”; insert “County” after “Butler”; change the “[20]” citation to “[22]” | This has been addressed. | 103-105 |
| Figure 1: suggest making county names more readable (move them on top of the well locations?) | This has been addressed. New map image included with submission. | NA |
| **Cumulative Well Density and Inverse Distance Weighting** |  |  |
| Citation numbering got messed up in a few spots. I think [20] in the first paragraph should be [21], and in the second paragraph [22] should also  be [21] while [20] should be [22]. Please check. Also in the second paragraph, I think you should insert “[19]” after the Weinberger citation. | This has been addressed. | 111,113,115 |
| Line 120: change “1km” to “1 km” (insert space) | This has been addressed. | NA – text modified or removed |
| Line 126: the “IDW” abbreviation was already established earlier. | This has been addressed. | NA – text modified or removed |
| Line 127: should “qualifying” be “quantifying”? Leading into the next line, change “closer to the respondents’ home” to “closer to a respondent’s  home”. | This has been addressed. | NA – text modified or removed |
| Line 128: suggest updating this sentence to “The inverse distance of each well within 1-, 2, and 5-km radii of a residence was calculated, and those  values were summed into one IDW score per respondent, per radius, as shown in the following equation:” | This has been addressed. | NA – text modified or removed |
| Lines 132–133: change “respondents’ home, and *n* is the number of wells within the 5 km buffer” to “respondent’s home, and *n* is the number of  wells within the radius” | This has been addressed. | 127 |
| **Ambient Air Emissions** |  |  |
| Line 139: why does it matter that emissions data after December 31, 2017 were unavailable? The health survey data stopped after 2017 anyway. | This has been addressed. | NA – text modified or removed |
| Line 140: insert hyphen: “emissions-inventory data” | This has been addressed. | 135 |
| Line 141: define PM 2.5 | This has been addressed. | 136 |
| Line 142: reference [22] should be [21] | This has been addressed. It is back to being [22] simple to do the removal and addition of citations in the reference list, but [22] reflects the citation for the PA DEP data. | 137 |
| Lines 144–146: what emission sources weren’t included? You mention this much later, but would be good to mention here, too. | This has been addressed. | 141-142 |
| Line 154: “that was collected at the Pittsburgh Allegheny County Airport” is more clearly stated as “for the Pittsburgh Allegheny County Airport” | This has been addressed. | 157 |
| Line 156: your study was through 2017, not 2016. | This has been addressed. | 162 |
| Line 162: insert hyphen: “16-km radius” | This has been addressed. | NA – text modified or removed |
| **Statistical Analysis** |  |  |
| Line 181: insert comma after “intensity of each exposure” | This has been addressed. | 190 |
| Line 185: insert “for” after “were used”? | This has been addressed. | 195 |
| Line 186: change “a model selection and averaging tool” to “a tool for model selection and averaging” | This has been addressed. | 196 |
| Line 187: change “best fit” to “best-fit” | This has been addressed. | 198 |
| Line 189: change “one hundred” to “100” | This has been addressed. | 200 |
| Line 190: I’m not sure what “to model assessment” means, and why 100  models? | 100 is the default number of models that are run prior to the fitting of the best models. Out of 100 models, the model with the lowest AIC value is considered the best combination of variables. | 205-206 |
| Line 194: change “model” to “models” | This has been addressed. | 204 |
| Line 202: insert comma after “emissions” | This has been addressed. | NA – text modified or removed |
| **Symptom Reporting Characteristics** |  |  |
| Line 227: is the median age better stated as “57 ± 1 standard deviation (SD)”? Note to put a space after ±. If this is the correct expression, then consider also updating lines 230–231 to say “with a mean of 7 ± 7.7 SD”. | This has been addressed. | NA – text modified or removed |
| Lines 229–230: is this sentence saying that some respondents reported 0 symptoms, while one or more respondents reported 36 symptoms, with an average of 7 reported per person? Consider rewording to be more clear. | This has been addressed. | 241-243 |
| **Generalized Linear Models: Symptom Total** |  |  |
| First sentence: consider rewording to “Based on the initial GLMs (including demographic variables), models using a 5-km radius for  cumulative well density had the lowest AIC value (relative to the 1- and 2-km radii), and 5 km was therefore used as the defining radius for  cumulative well density as an exposure variable…” | This has been addressed. | NA – text modified or removed |
| Line 238: change “from 0.05 to 0.73, thus all three” to “from 0.05 to 0.73; thus, all three” | This has been addressed. | 247 |
| Figure 2a’s X axis should have a space between 5 and km (“5 km”). | This has been addressed. | NA |
| Line 248: insert “did” before “emissions” | This has been addressed. | NA – text modified or removed |
| Line 250: revise to “In the cumulative-well-density model…” | This has been addressed. | NA – text modified or removed |
| **TITAN Analysis** |  |  |
| Lines 264–265: update “along the cumulative well density, IDW, and emissions gradients” to “along gradients of cumulative well density, IDW, and emissions”. Otherwise, proper grammar would suggest that you say “along the cumulative-well-density, IDW, and emissions gradients”, which is fine but some consider to be awkward. Consider this throughout this section (e.g., like 267 “cumulative-well-density gradient” or “gradient of cumulative well density”). | This has been addressed. | Language corrected starting at 262 and going through the “TITAN Analysis” section. |
| Figure 3 discussion: Suggest you introduce the figure on line 266: “Fig 3 shows results for cumulative well density, with the 23 significant  symptoms displayed. Itchy or burning eyes…”. Then later on line 270, you can remove the “Of the twenty-three significant symptoms, ” preface and just begin “Roughly 26% of the symptoms were categorized…”. | This has been addressed. | 269 |
| Lines 272–273: What is the meaning of negative associations? Increasing well density leading to decreasing reports of headaches, difficulty  speaking, ringing in ears, and rash? That’s odd, isn’t it? It might be worth mentioning your “type-I error” hypothesis here? Earlier on line 266, you  when discussing the top three indicator values, you probably should include that they’re positive associations, just to be clear? | These negative associations are considered type-I errors in our analysis. It does not necessarily mean that was density increased headache decrease, but rather we expect with so many symptom variables being thrown into the model, some will return as anomalies/errors. | 271-274  369-373 |
| Lines 272 and 281: should you use “negatively associated” or “inversely associated”? | This has been addressed. | NA – text updated in multiple places in section |
| Lines 284–285: maybe you mean “followed by nerves and muscle symptoms and psychological symptoms, which comprised 21% of  symptoms each”? | This has been addressed. | 286 |
| Lines 285–286: This sentence might be better as “In addition to headaches, difficulty speaking was also negatively associated with the gradient.”? Similarly, lines 294–295: “In addition to headaches, rash and palpitations were also negatively associated with the gradient.” | This has been addressed. | 282 |
| Lines 298–299: end of sentence might be better stated as “with psychological symptoms and nerve and muscle symptoms each at 20%.”? | This has been addressed. | 299 |
| **Discussion** |  |  |
| Lines 309, 314, 318, and 323: “human-health standpoint”, “human-health symptoms”, “human-health impacts”, “human-health metrics” (insert hyphen) | This has been addressed. | NA – text updated in multiple places in section |
| Line 311: I’m not 100% certain but I think “further” should be “farther”? | This has been addressed. | 311 |
| Line 314: change “non-UOGD” to “not UOGD” | This has been addressed. | 314-315 |
| Line 320: remove “that” | This has been addressed. | NA – text modified or removed |
| Line 322: change “does” to “do” | This has been addressed. | NA – text modified or removed |
| Line 325: “5-km” (insert hyphen) | This has been addressed. | 326 |
| Line 334: change “was” to “were” | This has been addressed. | NA – text modified or removed |
| Lines 335–336: “exposure-magnitude impacts (insert hyphen) | This has been addressed. | 343-344 |
| Line 346: I think the “which may lead to underreporting of impacts to health across the literature” should be bounded by commas | This has been addressed. | 351-352 |
| Lines 351–352: consider changing “in each model” to “in each of our models” for clarity | This has been addressed. | 357 |
| Lines 365–366: consider “related to the gradients of cumulative well density, IDW, and emissions, respectively”, rather than “related to the cumulative well density, IDW, and emissions gradient respectively”. Similarly later on line 370:  “associated with gradients of cumulative well density, IDW, and emissions, respectively, which contributes” | This has been addressed. | 355, 370 |
| Last sentence: why would these aspects lead to underestimates of emissions? | This has been addressed. | 398-402 |
| **Limitations & Recommendations** |  |  |
| Lines 375–376: “health-reporting data” (insert hyphen) | This has been addressed. | NA – text modified or removed |
| Line 378: should the Tustin and Rabinowitz citations have numbered indicators  (and Elliot on line 379, Rabinowitz on line 385)? | This has been addressed. | NA – text modified or removed |
| Lines 391–392: consider revising sentence to “In future studies, other health indicators or metrics such as blood pressure, heart rate, and the number of days a symptom persisted could provide a more in-depth analysis and help define the severity of the symptoms experienced.” | This has been addressed. | NA – text modified or removed |
| Line 400: change to “The air-and-exposure screening model” (insert hyphens) | This has been addressed. | 398 |
| **Conclusion** |  |  |
| Line 408: remove extra space before “an IDW metric” | This has been addressed. | NA – text modified or removed |
| Line 410: “detrimental health” is awkward, do you mean health complications? | This has been addressed. Yes, health complications. | 409 |
| Line 414: insert “the” before “relatively young age” | This has been addressed. | 412 |
| Line 416: change “but do raise” to “but they do raise” | This has been addressed. | 415 |
| Line 418: change “oil and gas industry” to “oil-and-gas industry” | This has been addressed. | NA – text modified or removed |
| Lines 418–419: suggest rewording to “Our study suggests that the narrow consideration of exposure risk used in some locations—based only on proximity to an individual well—may warrant revision to assess the contribution of the  growing density of wells in and around communities at 5-km scales.” | This has been addressed. | 416-420 |
