# Supplementary material for: Exposure assessment of adults living near unconventional oil and natural gas development and reported health symptoms in southwest Pennsylvania, USA
Source: PLoS One. 2020 Aug 18;15(8):e0237325. doi: 10.1371/journal.pone.0237325 (PMC7446921; doi:10.1371/journal.pone.0237325)
Supplement: Attachment — Submitted filename: response to reviewers PONE-D-19-34629.docx [file pone.0237325.s005.docx]

| **Comments** | **Response** |
| --- | --- |
| **Reviewer 2** | |
| 1. Why does the present study omit formaldehyde but it was included in the earlier study? | Brown et al. (2019) calculated emissions from wells, processing plants, and compressor stations. Brown et al. established the 5 compounds with the highest reported mass and known health effects. This analysis calculated emissions from wells only; this analysis did not include formaldehyde because it was not one of the top 5 compounds emitted from wells.  See lines 140-147 |
| 2. The final sample size ended up being 87 in the 2019 paper, were you able to acquire additional data? | The 2019 paper excluded those whose “residence was outside Pennsylvania and whose residence was outside of the county of interest”. In this paper, we excluded those whose residence was “outside of Pennsylvania” and included those residing in Washington, Greene, Beaver, Butler, Allegheny, Bedford, Fayette, and Westmoreland counties which resulted in a larger sample size.  See lines 87-100 |
| 3. What is the distribution of years that the participants took the survey in the present study? No emissions data was available in 2017, so did these subjects receive estimates from 2016? | Participants were distributed among years, with 23, 9, 29, 20, and 17 in the respective years between 2012-2016. The 2016 emissions data were used for the 6 participants from 2017.  See lines 119-120 & 179-180 |
| 4. Switched from 2 to 5km buffer between the two studies, what was the rationale? | In our first submitted manuscript, we included text in the methods “We applied GLM analyses using three spatial scales of cumulative well density: 1, 2, and 5 km. AIC criterion was used to determine the appropriate spatial scale to study” and in the results “Initial GLMs for cumulative well density at the three spatial scales against symptom total, including demographic variables, showed that models using cumulative well density in 5 km had the lowest AIC value and was therefore used as the defined radius in cumulative well density as an exposure variable (1 km: AIC=1095.26, 2 km: AIC=1039.73, 5 km: AIC=1024.86).”  We have included this text back into the manuscript to explain why 5 km was chosen.  See lines 216-218 & 254-256 |
| 1. Please add some explanation regarding the decision to combine 80+ (in many cases completely unrelated) symptoms into a single symptom count variable for the regression analysis. | The health assessment was conducted using a standard clinical interview, which included a comprehensive list of symptoms (such as one might see at any clinical visit with a health care provider). We have edited lines 81-94 to more accurately reflect the process. The number of symptoms reported ranged from 0-36, as stated in the results on line 249-250. Other studies have used this approach—i.e., counting total number of symptoms reported as an indication of health (Rabinowitz for example). It is important to note that it is not unreasonable to expect emissions of several compounds, each with specific health effects, might cause multiple body systems to be affected. |
| 2. How does the present study compare to Brown et al. 2019 where only respiratory symptoms were assessed and no association was found with air emissions? Please add some text to the discussion section on this topic. | This was added to the text:  Brown et al. 2019 did not find an association with the median air emissions. The most likely explanation for this inconsistency is that for this study, we used the 90^th^ percentile, rather than the median.  See lines 336-338 |
| 3. The figures continue to be too low resolution to really read. Please update these to at least 300 dpi. | We have used the PACE tool to provide the figures that meet journal requirements. Please download them and view on your computer to see if that enhances the quality compared to what is shown in the PDF. |
| 4. Add n (%) to table 1, please. | Column added to table. |
| 5. Discussion, page 21, line 429+: the CWD metric is most strongly associated with total symptom count. To me, this indicates a physical and psychological pathway between UNGD and health. Some discussion of how perceived environmental change is associated with health might be helpful here. For example, the idea of solastalgia, feeling homesick even at home due to changed environmental conditions around the home (Albrecht). Further, in Lai 2017 “Understanding the psychological impact of unconventional gas developments in affected communities,” the authors find that negative perceptions of unconventional development was associated with negative psychological states. | Thank you for this suggestion, we have edited the text  See lines 371-375 |
| 6. It should be emphasized that this study provides evidence that among people aware and concerned about UNGD the CWD metric performs best, we have no idea if this would be the case with people not concerned or pro-UNGD. There is an interplay of perception, psychology, and health that should be more carefully discussed. | While it is true that our sample was aware of/concerned for their health, they did not know about the exposure metrics our study explored nor that their symptoms would be looked at in this manner. It is safe to assume that the bias in our sample is minimized by that fact. Our exposure metrics were determined and designed years later. We assume respondents could not have over or under reported symptoms related to the exposure metrics given they did not know them. Additionally, we do not know if people who are concerned vs. not concerned (supportive vs unsupportive) are different from each other since we did not test our metrics with individuals who were not concerned about UOGD.  See lines 84-85, 245, & 389-399 |
| **Reviewer 2** | |
| Line 39 - remove “of” before “50%” | Addressed |
| Line 40 - change “grouping” to “groupings” | Addressed |
| Line 51 - remove hyphen in “human-health” | Addressed |
| Lines 62-63 - think you mean “Which exposure measure(s) is the best predictor…” or “Which exposure measures are the best predictors…” | Addressed |
| Line 76 - did you mean “different” instead of “difference”? | Addressed |
| MAJOR COMMENT: Lines 98-99 - it’s not clear why your analysis was restricted to the eight counties | Analysis was restricted to PA residents (who happened to be from just 8 counties) because we used PA DEP emission data.  See lines 96-100 |
| Line 116 - change “were plotted” to “was plotted” | Addressed |
| Line 119 - remove “was completed” | Addressed |
| Line 136 - change “rate” to “rates” | Addressed |
| Line 143 - you mean “concentrations” not “emissions” | Addressed |
| Lines 146, 147, 151 - “down-wind” “downwind” - choose one | Addressed |
| MAJOR COMMENT: Line 156 - I still don’t understand what part wind direction played in your modeling | The box model does not take wind direction into account. Brown et al. 2019 examined wind direction data from NOAA and found that the wind blows from the north, south, and west 90% of the time, with 10% coming from the east.  The use of putting sources in cardinal directions from a home was a broad way to think about wind direction but did not actually use the NOAA wind direction data in the analysis. This was updated in the text.  See lines 148-180 |
| MAJOR COMMENT: Line 159 - I still think you don’t adequately address in the paper the representativeness of the data at the Pittsburgh airport to the region at large. I don’t think it’s the only hourly station in the eight-county area that reports the variables you use. Why did you use this one station? | The approach we took was to choose one major airport where we felt the most complete set of weather data could be gathered and generalized over an area. Data from smaller airports is frequently incomplete even if they are closer to a home. We recognize this is a limitation of this exploratory study.  See lines 162-168 & 416-417 |
| Line 162 - change “was” to “were” | Addressed |
| Line 163 - remove “yearly” as it implies annual-average weather, when in fact you used hourly weather data | Addressed |
| Line 172 - change “respondents’” to “respondent’s” and “was” to “were” | Addressed |
| MAJOR COMMENT: Paragraph beginning Line 173 - Your methodology treats the trajectory of each well’s plume equally when summing the quadrant concentrations together. In fact, a wind rose analysis would tell you how often winds blew from each quadrant toward each residence, allowing you to weight the concentrations from some quadrants more than others. You should either address this limitation or correct for it. | We have addressed this in the limitations section.  See lines 411-414 |
| Line 173 - suggest changing “surround” to “are ubiquitous around” | Addressed |
| Line 176 - I think you mean “in which quadrant the well was located relative to the residence”? | Addressed |
| Line 182 - change “of their survey and was used” to “of their survey, which was used” | Addressed |
| Line 186 - should “compared” be changed to “made”? | Addressed |
| Line 201 - should “to generation” be “for determining”? | Addressed |
| Line 213 - “a species” is singular but “taxa” is plural - change “taxa” to “taxon”? | Addressed |
| Line 214 - change “are” to “is” | Addressed |
| Line 223 - change “uses” to “used” | Addressed |
| Line 232 - change “z score” to “z-score” | Addressed |
| Line 248 - change “measures” to “measure” | Addressed |
| I don’t think Figure 2 is cited in the text? | Addressed |
| Lines 306, 314 - remove hyphen in “human-health” | Addressed |
| MAJOR COMMENT: Line 311 - you only evaluated wells within 5 km of a residence, so how did you detect effects beyond that? | We are stating that our study used a further range than references like [15,19] and detected effects beyond where those researchers studied. We did not look beyond 5 km. The text has been modified to help clarify that statement.  See lines 318-321 |
| Line 333 - remove “primarily”, all respondents were concerned about UOGD exposure | Addressed |
| Line 342 - end of line, change “was” to “were” | Addressed |
| Line 343 - remove hyphen in “exposure-magnitude” | Addressed |
| Line 395 - I believe development and production are the ONLY UOGD stages, and I believe the literature supports that emissions from development tend to be higher than those from production | Addressed |
| MAJOR COMMENT: Line 401 - your model actually does account for day-to-day weather patterns, as it utilizes hourly weather data. Do you mean that your model does not correct for differences in weather observations at residences relative to the weather station site? | Yes, that is correct, the model does account for day to day weather patterns each day of the year. We have removed that line from the text. The main limitation is that the box model does not correct for topography. |
| Line 406 - I think you mean “emissions concentrations”. Air dispersion modeling does not quantify emissions, it uses them to quantify concentrations. Also, your study is not the first to use dispersion modeling for UOGD purposes. | Addressed |
| Line 411 - remove hyphen in “human-health” | Addressed |
| Lines 417-418 - I think you want to remove “in and around communities at” | Addressed |
| Line 419 - remove hyphen in “residence-level” | Addressed |
| Line 420 - remove hyphen in “human-health” | Addressed |
| Line 577 - change “CDW” to “CWD” | Addressed |
