# Supplementary material for: Exposure assessment of adults living near unconventional oil and natural gas development and reported health symptoms in southwest Pennsylvania, USA
Source: PLoS One. 2020 Aug 18;15(8):e0237325. doi: 10.1371/journal.pone.0237325 (PMC7446921; doi:10.1371/journal.pone.0237325)
Supplement: Attachment — Submitted filename: PONE-D-19-34629_R2_Review.docx [file pone.0237325.s006.docx]

Thank you for the opportunity to again review your paper. I appreciate that you have addressed all prior comments. However, I have significant reservations on the 2016/2017 mismatch of data—this is highlighted below. I will not recommend for publication without addressing this issue.

I will be happy to review your next revision, should you decide to pursue that.

**Introduction**

- Line 51: change “adverse health” to “adverse health effects” (or outcomes, etc.)

**Materials and Methods**

**Study Sites & Health Outcomes**

- Line 85: data is plural, so change “was abstracted” to “were abstracted”
- Lines 95–96: convert “General” to lower case; suggest using semicolons in the list so that the EENT group is separated more clearly from skin; also, need to put the EENT group in the right order. So: “general; lung and heart; skin; eyes, ears, nose, and throat; gastrointestinal (GI); nerves and muscle; reproductive; blood system; and psychological”
- Line 99: 14 individuals were excluded because you didn’t know their home lat/longs right? If so, move the parenthetical to the end of the previous sentence.
- Lines 105–106: If no respondents lived in Lawrence County, then why color it dark gray indicating “county with study residencies”? (did you mean “residences”?) Why does it matter that someone in Butler County lived near the Lawrence border?

**Exposure Measures**

**Cumulative Well Density and Inverse Distance Weighting**

- **MAJOR COMMENT** Lines 119–120 (and 168): You used 2016 well and weather data for comparison to 2017 health survey data? How is that appropriate? Your data are already of low temporal resolution (one-time reporting of symptoms related to annual well data), and with this mismatching of 2016/2017, I find myself wondering how much different your study outcomes would be if you just randomized which year of data were applied to each respondent. You need a discussion of this in your discussion/limitations, or remove 2017 from your assessment, or redo the 2017 assessment with 2017 well/weather data if available.

**Annual Emissions Concentration**

- Lines 148–149: remove either “were derived” or “were estimated”, it’s redundant
- Line 150: I think you should use “respondent’s” instead of “participant’s” for consistency.
- Line 165: “hourly conditions for each hour” is redundant

**Statistical Analysis**

- Line 208: change “determining” to “determine”

**Results**

**Generalized Linear Models: Symptom Total**

- Line 255: change “and was therefore selected” to “and were therefore selected”

**Discussion**

- Line 316: alter to “Despite a high degree of inherent complexity in associations between health and UOGD…”
- Line 328: change “attempt” to “attempts”
- Lines 354–356: this is an incomplete sentence

**Limitations & Recommendations**

- Line 397: I think you should use “respondents” instead of “participants” for consistency.
